# Impact of gene therapy for canine monogenic diseases on the progress of preclinical studies

**DOI:** 10.1007/s13353-020-00554-8

**Published:** 2020-03-18

**Authors:** Marek Switonski

**Affiliations:** Department of Genetics and Animal Breeding, Poznan University of Life Sciences, Poznan, Poland

**Keywords:** Dog, Human, Retinal diseases, Lysosomal storage diseases, Immunodeficiency, Leukocyte adhesion deficiency, Hemophilia, Muscular dystrophy

## Abstract

Rapid progress in knowledge of the organization of the dog genome has facilitated the identification of the mutations responsible for numerous monogenic diseases, which usually present a breed-specific distribution. The majority of these diseases have clinical and molecular counterparts in humans. The affected dogs have thus become valuable models for preclinical studies of gene therapy for problems such as eye diseases, immunodeficiency, lysosomal storage diseases, hemophilia, and muscular dystrophy. Successful gene therapies in dogs have significantly contributed to decisions to run clinical trials for several human diseases, such as Leber’s congenital amaurosis 2—LCA2 (caused by a mutation of *RPE65*), X-linked retinitis pigmentosa—XLRP (caused by mutation *RPGR*), and achromatopsia (caused by mutation of *CNGB3*). Promising results were also obtained for canine as follows: hemophilia (A and B), mucopolysaccharidoses (MPS I, MPS IIIB, MPS VII), leukocyte adhesion deficiency (CLAD), and muscular dystrophy (a counterpart of human Duchenne dystrophy). Present knowledge on molecular background of canine monogenic diseases and their successful gene therapies prove that dogs have an important contribution to preclinical studies.

## Introduction

The dog is an exceptional species in terms of the phenotypic variability observed in the approximately 400 breeds recognized by the Fédération Cynologique Internationale (FCI) and the American Kennel Club (AKC). The majority of breeds were created over the last two centuries, despite the fact that dogs have been domesticated since approximately 15,000 years ago (vonHoldt et al. 2010; Ostrander et al. 2017). The various breeds were usually derived from a small number of founders, followed by stringent selection for desired traits, sometimes interrupted by bottleneck events that created unique breed-specific gene pools. These pools, besides the variants responsible for the desired traits, also contained undesired mutations causing monogenic diseases, as well as variants predisposing to diseases with complex backgrounds (such as cancer, obesity, and hip dysplasia). It is therefore unsurprising that the dog is considered an important large animal model that is useful in preclinical studies of human hereditary diseases, including gene therapy (Shearin and Ostrander 2010; Switonski 2014). Approximately 700 monogenic diseases have been described in dog breeds, including at least 230 with known causative mutations. About 430 of these are considered potential models for human diseases (January 20, 2020, https://omia.org/home).

Knowledge of the molecular background of a broad range of hereditary canine diseases was developed on the basis of extensive studies of the dog genome, which begin in 1993 during the First International DogMap Meeting in Oslo, Norway (Report from the First International DogMap Meeting 1993). The main goals of this international collaboration were to establish linkage and cytogenetic (mainly based on microsatellites) marker maps of the dog genome (Dolf et al. 1999, Switonski et al. 2004). The next step was genome sequencing of the dog, initially among domestic mammals (Kirkness et al. 2003, Lindblad-Toh et al. 2005). It was found that the genome’s total length, at about 2.4 Gb, is shorter than the human genome, at around 2.9 Gb. The estimated number of protein coding genes was also slightly lower at approximately 19,300. The third step in canine genome studies—the analysis of genome sequence variation in 10,000 dogs of different breeds—is presently being undertaken (Ostrander et al. 2019).

Knowledge of marker maps and nucleotide sequences of the dog genome, along with the availability of new molecular tools (SNP microarrays, NGS, and others) has facilitated deep insight into the molecular background of breed-specific phenotypes. It is well known that some of these traits are in fact hereditary congenital disorders whose molecular background has been elucidated, as is the case with brachycephaly (Marchant et al. 2017) and chondrodysplasia (Parker et al. 2009; Brown et al. 2017),

Clinical trials of gene therapy have been performed since 1989, and from then until 2017, at least 2600 trials have been undertaken. The majority of these (65%) dealt with cancers, and monogenic disease were studied in only 11% of trials (Ginn et al. 2018). These clinical trials resulted in the approving of almost twenty recombinant products for gene therapy in human medicine (Shahryari et al. 2019).

Clinical and molecular characterization of numerous canine monogenic diseases was the first step towards using affected dogs in preclinical studies of gene therapy. The first successful gene therapies in dogs for monogenic diseases were reported in the middle 1990s of the twentieth century (Table 1). Since then, numerous studies for different diseases have been published, including long-term follow-up of the treated dogs. In this review, the significance of these preclinical studies for human gene therapies is outlined.

**Table 1:** First successful attempts of gene therapy for selected canine monogenic diseases

Year	Gene therapy in dogs				Human counterpart disease
	Disease	Corrected gene	Vector*	Reference	
1993	Hemophilia B	*FIX*	RV	Kay et al. 1993	Hemophilia B
1996	Hemophilia A	*FVIII*	AV	Connelly et al. 1996	Hemophilia A
1998	Muscular dystrophy	*DMD*	AV	Howell et al. 1998	Duchenne muscular dystrophy
2001	Congenital stationary night blindness (CSNB)	*RPE65*	AAV	Acland et al. 2001	Leber amaurosis type 2 (LCA-2)
2002	Mucopolysaccharidosis VII (MPSVII)	*GUSB*	RV	Ponder et al. 2002	Mucopolysaccharidosis VII
2006	X-linked severe combined immunodeficiency (X-SCID)	*IL2RG*	RV	Ting-De Ravin et al. 2006	X-linked Severe Combined Immunodeficiency (X-SCID)
2008	Canine leukocyte adhesion deficiency (CLAD)	*ITGB2 (CD18)*	FV	Bauer et al. 2008	Leukocyte Adhesion Deficiency 1 (LAD-1)
2010	Achromatopsia	*CNGB3*	AAV	Komáromy et al. 2010	Achromatopsia
2012	X-linked retinitis pigmentosa	*RPGR*	AAV	Beltran et al. 2012	X-linked retinitis pigmentosa

*AV adenovirus, *AAV* adeno-associated virus, *RV* retrovirus, *LV* lentivirus, *FM* foamy virus

## Eye diseases

Monogenic retinal diseases have been diagnosed in numerous dog breeds, and causative mutations have been identified for many of them (Mellersh 2014). A large group of these diseases are classified as progressive retinal atrophy (PRA), a counterpart of human retinitis pigmentosa (RP). Mutations in seventeen canine genes have been recognized to date as the cause of different types of PRA, including rod–cone dysplasias (RCD), progressive retinal atrophies (PRA), generalized progressive retinal atrophy (GPRA), and progressive rod–cone degeneration (PRCD). Canine PRAs are thus considered natural models of human RP (Petersen-Jones and Komáromy 2015; Bunel et al. 2019).

The most spectacular success seen in gene therapy of canine ocular disease is related to congenital stationary night blindness (CSNB), a counterpart of type-2 Leber’s congenital amaurosis (LCA2) in humans. CSNB in Briard dogs is caused by a recessive mutation of *RPE65* gene (Aguirre et al. 1998). Mutations of this gene are also responsible for human LCA2 (Kondkar and Abu-Amero 2019). Soon after the discovery of the causative mutation of CSNB, successful gene therapy employing a complementary sequence of the *RPE65* gene carried by an adeno-associated virus (AAV-RPE65) was reported by Acland et al. (2001). A subretinal injection of the gene construct was given to three dogs, all of whom regained their vision. Further studies have shown that this therapy was successful in 23 of 26 treated eyes, and that the therapeutic effect was stable for at least 3 years (Acland et al. 2005). The first human clinical trial for LCA2 gene therapy was performed in three adult patients (19, 26, and 26 years old) (Maguire et al. 2008). The researchers emphasized that the preclinical study carried out on the dog model was a very important step leading to the clinical trial. The trial showed a modest improvement in retinal function. Later clinical trials also confirmed that the improvement in visual function is safe and stable (Testa et al. 2013; Schimmer and Breazzano 2015). These reports and earlier preclinical studies enabled the Food and Drug Administration (FDA) to approve this gene therapy for common use in December 2017 (https://www.fda.gov/media/109487/download). This was the first gene therapy for monogenic disease approved in the USA. A recent study carried out on affected dogs given gene therapy at an age of 5–6 years showed that its efficacy is positively correlated with the proportion of photoreceptors remaining at the time of the treatment. It was found that, in retinal regions with a lower proportion of normal photoreceptors (< 63%), degeneration progresses similarly to untreated regions. It is therefore expected that gene therapy may not stop the progressive degeneration of the entire retina (Gardiner et al. 2020).

Successful gene therapy has also been reported for canine X-linked retinitis pigmentosa (XLRP), which is caused by a mutation of the retinitis pigmentosa GTPase regulator (*RPGR*) gene (Beltran et al. 2012). The researchers used an AAV vector carrying cDNA of a functional fragment of the human *RPGR* gene. Subretinal injection of the construct resulted in a long-lasting (> 6.5 years after the treatment) preservation of retinal function (Beltran et al. 2019). Successful preclinical studies carried out on murine and canine models are currently being followed up on by phase I, II, and III clinical trials (Cehajic Kapetanovic et al. 2019).

The most common form of human RP disease is caused by dominant mutations of the rhodopsin (*RHO*) gene (Athanasiou et al. 2018). This type of RP has also been diagnosed in English Mastiff dogs (Kijas et al. 2002). An efficient approach based on the use of a recombinant AAV vector with dual functions has recently been used (Cideciyan et al. 2018). The injected vector had the following functions: (*) suppression of normal and mutated *RHO* gene by a short hairpin RNA (shRNA) and (**) expression of human cDNA of the *RHO* gene that was resistant to shRNA.

Achromatopsia is a group of rare human diseases manifesting as abnormal cone photoreceptor function; it can be caused by mutations of several genes (Hassall et al. 2017), including of *CNGB3*, which encodes cyclic nucleotide–gated channel beta 3. Different causative mutations of this gene have been identified in several breeds, including Alaskan Malamute, German Shorthaired Pointers, Miniature Australian Shepherd, Siberian Husky, and Alaskan Sled Dog (Dixon 2016). Two mutations have been successfully treated by subretinal injection of an AAV vector with human *CNGB3* gene (Komáromy et al. 2010). Again, positive results in preclinical studies carried out on mouse and dog models have enable clinical trials (Michalakis et al. 2017).

## Lysosomal storage diseases

Human lysosomal storage diseases (LSD) make up over 70 monogenic diseases, whose cumulative incidence is approximately 1 in 5000 live births (Platt et al. 2018). Numerous LSDs have also been diagnosed in dog breeds, of which mucopolysaccharidoses (MPS) and neuronal ceroid lipofuscinoses (NCLs) are the most interesting models for preclinical studies. Causative mutations have been identified for eight NCLs (Katz et al. 2017) and five MPS (Switonski 2014) diseases.

Preclinical testing of gene therapy has been used in four canine MPS diseases: MPS I, MPS IIIB, MPSVI, and MPSVII (Bradbury et al. 2015). The first gene therapy attempt was targeted to canine MPS VII, which is caused by mutation of the *GUSB* gene encoding β-glucuronidase enzyme (Ponder et al. 2002). This type of MPS is very rare in humans (Khan et al. 2017). The long-term outcome of this therapy in dogs was evaluated in terms of skeletal, heart, and neurological abnormalities. The effects of intravenous injection of gamma retroviral vector expressing *GUSB* gene in affected puppies was analyzed 10 years later, and it was found that the treated dogs could still walk, while untreated animals could not stand beyond 6 months and died at the age of 2 years. However, some abnormalities (osteophyte formation, cartilage, and gait abnormalities) were not corrected by the therapy (Xing et al. 2013). A positive long-term effect of this therapy was also observed for cardiac valve disease (Bigg et al. 2013). Since MPS VII also affects the central nervous system, the efficiency of the intravenous injection of the recombinant AAV vector was compared with that of intrathecal delivery; it was found that the latter was much more efficient (Gurda et al. 2016). This observation showed that intravenous delivery of the vector is alone not sufficient to achieve positive therapeutic effect in the nervous system.

Neuronal ceroid lipofuscinoses (NCLs) occur in human populations at low frequency and usually oscillates around 2 per 100,000 (Nita et al. 2016). Interestingly, these diseases are not very rare in dogs. They have been diagnosed in eleven dog breeds, and thirteen mutations responsible for eight types of NCL have been identified (reviewed by Katz et al. 2017). Canine NCLs are thus important targets for preclinical studies.

To date, 27 completed or ongoing clinical trials of gene therapy for LSDs—including 13 for MPS—have been undertaken. In addition, at least 29 promising preclinical studies have been carried out, including canine MPS I, MPS IIIB, MPS VII, and Krabbe disease (Nagree et al. 2019).

## Severe combined immunodeficiency diseases and leukocyte adhesion deficiencies

Severe combined immunodeficiency diseases (SCIDs) are rare monogenic diseases in humans, caused by mutations of nineteen genes. The overall incidence of these diseases in human populations varies widely, from 1 in 5000 (Saudi Arabia) to 1 in 78,000 newborns (Chile) (Kelly et al. 2013).

Mutations of three genes causing canine SCIDs have been identified. These genes are also responsible for human SCIDs. An autosomal SCID caused by mutation of the *DNA-PK* gene encoding DNA-dependent protein kinase was diagnosed in Jack Russell Terriers; X-linked SCID (X-SCID), caused by two different mutations of the *IL2RG* gene (encoding interleukin 2 receptor subunit gamma) specific to the Basset Hound and Cardigan Welsh Corgi breeds were reviewed by Perryman (2004). Another type of autosomal SCID, also known in humans and caused by a mutation of the *RAG1* gene encoding recombination activating protein 1, was diagnosed in Frisian Water Dogs (Verfuurden et al. 2011). There have been no reports on canine ADA-SCID caused by mutation of adenosine deaminase gene, which is a quite common form of human SCID, whose prevalence among SCID patients may reach 15% (Flinn and Gennery 2018). It should be pointed out that this monogenic disease was the first for which gene therapy trial was performed. Long-term follow-up of numerous treated patients resulted in the European Medicines Agency approving this gene therapy in patients for whom bone marrow transplantation, due to a lack of a donor, is not possible (https://www.ema.europa.eu/en/medicines/human/EPAR/strimvelis).

There are three forms of leukocyte adhesion deficiency (LAD) disease; they are rare in humans, with an incidence of approximately 1 per 1,000,000 newborns (Almarza Novoa et al. 2018). In dogs, two forms of LAD (LAD I, also called canine LAD I (CLAD I), and LAD III or CLAD III) have been described. Type I, which is caused by a mutation of *ITGB2* encoding integrin subunit beta 2 (CD18), was detected in Irish Setters and the closely related Irish Red and White Setter breeds (Gu et al. 2004); type III, caused by a mutation of Kindlin 3 (also called *FERMT3*) and encoding kindling 3 protein (also called fermitin family member 3) has been described in German Shepherd and in a German Shepherd × Rottweiler mongrel (Hugo and Heading 2014).

Gene therapies have been performed for canine X-SCID and CLAD I. In vivo and ex vivo procedures using different viral vectors were successful for X-SCID (reviewed by Felsburg et al. 2015). The researchers concluded that “Canine XSCID provides a unique large animal preclinical model for evaluating methods for improving the efficacy and safety of gene therapy for human XSCID”*.* The application of gene therapy for CLAD I, using hematopoietic stem cells transduced with foamy viral vector harboring the CD18 gene construct, was successful, as documented by a long-term (up to 7-year) follow-up of four treated dogs (Bauer et al. 2013).

## Muscular dystrophy

Duchenne muscular dystrophy (DMD) is caused by mutations of the dystrophin gene, and is a quite common monogenic sex-linked human disease, whose incidence is approximately 1:5000 in newborn boys (Yiu and Kornberg 2015). The development of an effective therapy for this disease is a very important issue, and compensation of dystrophin deficiency by gene therapy is considered to be the most promising approach (Salmaninejad et al. 2018).

In dogs, this disease is also caused by mutations of the dystrophin gene, and has been diagnosed in eight breeds: Golden Retriever, Labrador Retriever, German Shorthaired Pointer, Rottweiler, Cavalier King Charles Spaniel, Pembroke Welsh Corgi, Cocker Spaniel, and Tibetan Terrier (reviewed by Kornegay et al. 2012). It is therefore not surprising that canine muscular dystrophy has become a valuable large animal model for testing different approaches to gene therapy. Progress in these therapies has been recently reviewed by Nghiem and Kornegay (2019). A recent and a very promising attempt was based on the use of CRISPR/Cas9 technology, which was used for the first time for gene therapy of this disease (Amoasii et al. 2018). The researchers used intramuscular or systemic delivery of the components for gene editing and observed dystrophin restoration in skeletal and cardiac muscles. Since the dogs were examined shortly after the treatment (at 6 or 8 weeks), a long-term follow-up is necessary to evaluate its efficacy and safety (Wasala et al. 2019).

## Hemophilia

There are two types of hemophilia, both of which are X-linked monogenic diseases. Hemophilia A is caused by mutations of the *F8* gene encoding coagulation factor VIII, while hemophilia B develops due to mutations of the *F9* gene, which encodes coagulation factor IX. The prevalence of these diseases in human populations is different and hemophilia A is more frequent, with between 7 and 13 affected persons per 100,000 males (Stonebraker et al. 2010); the frequency of hemophilia B is between 1 and 3 per 100,000 males (Stonebraker et al. 2012). Both forms of hemophilia have also been observed in dog breeds. Hemophilia A was diagnosed in Irish Setters, Miniature Schnauzers, and German Shorthaired Pointer and hemophilia B in Lhase Apso, Labrador Retriever, and a Cairn terrier × Beagle crossbreed (Lozier and Nichols 2013).

Both types of hemophilia have been targeted by gene therapy in the preclinical study and clinical trial stages (Batty and Lillicrap 2019). The preclinical studies were carried out on dogs and tested a range of vectors, including retroviruses, adenoviruses (AV), adeno-associated viruses (AAV), and lentiviruses (Nichols et al. 2016). Of these, the AAV vectors seemed most convenient. Successful preclinical studies were followed up by clinical trials for hemophilia A and B, using the same vector (Peyvandi and Garagiola 2019; https://hemophilianewstoday.com/gene-therapy/).

## Conclusion

The dog has become an important large animal model for preclinical studies of gene therapy for monogenic diseases, on account of several important advantages over classical rodent models. The major advantages of this model include the spontaneous occurrence of the causative mutations, the existence of detailed pedigree information, and the opportunity for long-term follow-up of the dogs treated with gene therapy. The importance of dog as the model organism for preclinical studies has been recently emphasized by the fact that a special issue of *Human Genetics* (vol. 128, issue May 2019) was devoted to “Canine genetics”.
